# Nanocomposites Based on Disentangled Ultra-High Molecular Weight Polyethylene: Aspects and Specifics of Solid-State Processing

**DOI:** 10.3390/polym16233423

**Published:** 2024-12-05

**Authors:** Oleg V. Lebedev, Ekaterina P. Tikunova, Tikhon S. Kurkin, Evgeny K. Golubev, Alexander N. Ozerin

**Affiliations:** 1 Laboratory of Structure of Polymer Materials, Enikolopov Institute of Synthetic Polymer Materials Russian Academy of Sciences (ISPM RAS), Profsoyuznaya St. 70, 117393 Moscow, Russiaozerin@ispm.ru (A.N.O.); 2 Moscow Center for Advanced Studies, Kulakova Str. 20, 123592 Moscow, Russia

**Keywords:** ultra-high-molecular-weight polyethylene, nanocomposite, solid-state processing, carbon nanoparticle, electrical conductivity

## Abstract

The stages of solid-state processing of nanocomposites, based on nascent disentangled ultra-high-molecular-weight polyethylene (d-UHMWPE) reactor powders (RPs) and carbon nanoparticles (NPs) of various types, were meticulously investigated. The potential for optimizing the filler distribution through variation of the processing parameters, and the impact of the d-UHMWPE RP and nanofiller type on the electrical conductivity of the resulting composites were discussed. The specifics of the dependences of conductivity and tensile strength on the deformation ratio for the composites, oriented under homogeneous shear conditions, were investigated. The obtained results and the results on piezoresistivity and temperature dependency of conductivity in the oriented and compacted composites demonstrated the independence of the UHMWPE matrix orientational strengthening on the filling. The interchangeability of high-temperature uniaxial deformation and deformation under homogeneous conditions for orientational strengthening and electrical conductivity changes in the preliminary oriented composite samples was confirmed. The potential for simultaneously achieving high strength and conductivity in composite tapes and the possibility of directly processing d-UHMWPE RP and NPs mixtures into oriented composite tapes were demonstrated. The overall results suggest that the studied composites may serve as a viable model system for investigating the deformational behavior of conductive networks comprising NPs of varying types and contents.

## 1. Introduction

Polymer nanocomposite materials are typically designed to exhibit properties and characteristics that are essential for their practical applications. These properties, such as high thermal or electrical conductivity, can be susceptible to external stimuli, making them valuable in the development of various sensors and devices, antistatic, electrostatic, and electromagnetic interference (EMI) shielding coatings and elements, and other applications [1,2,3]. In highly electrically conductive polymer composites the concentration of the electrically conductive filler is generally considerably higher than the corresponding percolation threshold value [4]. Given that the percolation threshold is typically reached at high degrees of filling, numerous challenges emerge with regard to the processability of polymer composites, the quality of products derived from these materials, and their ultimate cost [5]. To address this issue, it is possible to create in the composite volume a segregated structure, in which the regions of heightened filler concentration have the potential to form a percolation cluster [6].

Polymer composites with a segregated structure have been a popular topic of scientific interest in the last decades [7]. For example, in their recent publication [8], Kuang et al. demonstrated the possibility of the construction of a secondary electron path through the addition of expanded graphite onto the interface between polylactic acid particles filled with multi-walled carbon nanotubes (MWCNTs). The obtained composites possessed integrated EMI shielding and joule heating capabilities and were effectively used as triboelectric nanogenerators. Different bioelectronic applications of such composites were discussed, including sensors, health monitors, smartwatches, innovative smart textiles, etc.

Among the multitude of polymers utilized as a matrix to create polymer nanocomposites, ultra-high-molecular-weight polyethylene (UHMWPE) stands out due to its unique combination of useful performance characteristics [9]. These characteristics include low density, low friction coefficient, chemical resistance to aggressive gases and liquids, hydrophobicity, frost resistance, gamma radiation resistance, abrasion resistance, physiological inertness, etc. [10]. This has led to a keen interest among researchers in the development of methods for obtaining composite materials based on UHMWPE.

A number of publications describe a processing approach in which filler nanoparticles (NPs) are preliminarily distributed mechanically along the boundaries of UHMWPE reactor powder (RP) particles. This is typically followed by the hot pressing of the resulting mixtures [11,12], which allows the creation of a distinctive segregated conductive network made of the filler NPs, akin to a honeycomb structure. Such UHMWPE-based composites represent a particularly intriguing research subject. For example, in the work of Wei et al. [13], the findings of an investigation into the correlation between temperature and electrical conductivity in hybrid composites comprising polypropylene, UHMWPE, and carbon black (CB) particles are presented. The preliminary mixing of the components in a liquid medium using ultrasound, followed by hot pressing at 190 °C, resulted in the formation of a pronounced segregated structure of CB particles within the composite material. A change in the temperature coefficient of electrical conductivity was observed as the polymer components underwent melting. Similar results were obtained in the work of Zhao et al. [14] for the graphite/polyurethane/UHMWPE system.

In their work [15], Azam et al. presented the results of a study of mechanical and piezoresistive behavior under tensile, flexural, and cyclic loadings for UHMWPE-based composites filled with MWCNTs and processed via selective laser sintering. The obtained composites demonstrated a linear piezoresistive response of the 2D-hexagonal nanocomposite lattice and consistent and stable strain sensing capability over 100 repeated load cycles. These findings illustrate the potential of UHMWPE-based nanocomposites for the advancement of smart biomedical devices.

In the work of Mamunya et al. [16], the shielding efficiency of UHMWPE-based composite materials was attributed to the formation of a cellular structure in the bulk of the composite. These cells were composed of polymeric insulating grains covered with a filler layer, exhibiting a degree of variation in their sizes. The distribution of the filler in a thin layer on the surfaces of the polymer grains, as well as the distribution of electrically conductive cells in the bulk of the composites, resulted in a system comprising two levels of structuring. This enabled radiation entering the bulk of the composite to undergo multiple reflections from highly filled interlayers in the cellular structure, thereby achieving efficient absorption. In the work of Yu et al [17], the composites based on UHMWPE filled with MWCNTs were obtained through the mechanical mixing of components followed by hot pressing at 200 °C. The addition of MWCNTs to the composite resulted in an increase in shielding capacity in the transverse direction. Furthermore, orientational stretching of the material contributed to this increase in the shielding capacity. This phenomenon was attributed to the alteration in the cell period of the segregated structure in both the transverse and longitudinal directions, which can potentially lead to resonance at a specific frequency of electromagnetic radiation within the entire volume of the cells at a particular drawing ratio.

The close-to-extreme segregation of the filler in the bulk of composites based on UHMWPE results in an exceptional set of functional characteristics. For example, nanocomposites based on UHMWPE are readily utilized in the medical field as biocompatible implants [18], or as tribological components [19].

As demonstrated by Mohammed et al. [20], the incorporation of montmorillonite into UHMWPE can significantly reduce wear and the coefficient of friction. With an increase in the montmorillonite content in the composite, the wear and friction coefficient began to increase, indicating the necessity to optimize the degree of filling in order to obtain composites with the desired set of characteristics. In their study, Hui et al. [21] investigated the impact of NPs’ (graphene oxide) interaction on the barrier properties of UHMWPE composites. These composites were produced through hot pressing of polymer and filler particles. The results indicated that optimizing the degree of filling is crucial for enhancing the barrier properties of the composites.

In the work of Cheng et al. [22], the importance of filler–polymer interaction was discussed in the context of UHMWPE/CB composites, where the filler exhibited a highly segregated distribution within the polymer volume. It was shown that the modification of the filler can have a significant impact on the mechanical characteristics of the material. Overall, a notable decline in the strength of the composite was observed with an increase in the filler content.

To enhance the strength of UHMWPE-based composite materials, the orientational strengthening of the polymer matrix can be used [23]. However, the orientational deformation (drawing) of composite materials filled with highly dispersed NPs is frequently accompanied by brittle fracture at the initial stages of deformation [24,25,26]. In their work [27], Kechek’yan et al. described a solid-state (below the melting point of the polymer) approach for overcoming the brittle fracture of filled polymer nanocomposites during their orientational deformation. This was achieved through preliminary deformation under homogeneous shear conditions. After the treatment, the polymer composite material acquired the ability for high uniaxial orientation deformation. This solid-state orientational strengthening is possible only for nascent disentangled UHMWPE (d-UHMWPE) RP [28].

The term disentangled refers to UHMWPE with fewer entanglements in the amorphous regions, a metastable condition that can significantly affect the material properties and processing behavior [29]. Nascent d-UHMWPE RPs do not possess the intrinsic melting defects and crystal boundaries within the commercial UHMWPE [30]. The lower entanglement density in d-UHMWPE facilitates the solid-state processability into uniaxially-oriented specimens reaching very high deformation ratios and crystallinities [31]. The processed d-UHMWPE was found to demonstrate superior drawability, flexibility, toughness, and thermal conductivity, among other properties. It is used in a variety of applications, including packaging, bulletproof armor, joint replacements, aerospace, marine engineering, etc. [30].

In the work of Ronca et al. [28], it was shown that through the implementation of a controlled synthesis process, a diverse range of linear d-UHMWPE RPs can be produced, which can then be subjected to uniaxial stretching. It was previously discussed that the solid-state-processed tapes do not achieve the high values of tensile strength that would be expected from the theory. The relatively lower tensile strength observed in the uniaxially stretched tapes was attributed to the presence of defects, as tapes can be considered composites of fibers. It was also concluded that the high modulus in combination with the tensile strength in tapes has the potential to provide unique physical properties in UHMWPE-based composites.

In the work of Golubev et al. [32], structural studies were conducted and the characteristics of samples of UHMWPE were determined for successive stages of the continuous solid-state processing of nascent d-UHMWPE RPs into high-strength tapes. The processing stages included compacting, homogeneous shear deformation (calendering), and orientational stretching (drawing). The capacity of the d-UHMWPE RP to be effectively compacted into samples with satisfactory mechanical characteristics (tensile strength ~ 10 MPa) was attributed to the formation of molecular entanglements between adjacent crystallites during the compaction, thereby ensuring the subsequent effective monolithization of a compact sample through the application of homogeneous shear deformation [33]. It was demonstrated that the utilization of nascent d-UHMWPE RP with the optimal morphology, molecular weight, and bulk density for solid-state processing enables the production of tapes with high strength (>3 GPa) and high modulus (>120 GPa) [34,35]. The influence of the processing conditions, including those for both compacting/homogeneous shear deformation and orientational stretching, on the structure and properties of highly oriented tapes was investigated. It was determined that the processing parameters can be effectively optimized to prepare materials with a combination of the highest elastic strength characteristics.

In the works of Lebedev et al. [36,37], it was demonstrated that strengthened electrically conductive composites based on d-UHMWPE RP filled with such carbon NPs, such as CB, GNPs, and MWCNTs, can be obtained through solid-state processing of the mixtures of d-UHMWPE RP and NPs. The unoriented composite samples were produced by preliminary coating the d-UHMWPE RP particles with NPs, followed by cold compaction of the mixtures. Subsequently, the samples were deformed under homogeneous shear conditions until a specific deformation ratio was reached. Due to the solid-state nature of the processing, the filler NPs remained densely packed at the boundaries of the UHMWPE grains, unable to enter the polymer grain volume. Meanwhile, the zones of contact between NPs did not contain any polymer phase. This was considered as an example of the extreme segregation of the filler within a polymer composite volume.

The feasibility of multiscale modeling aimed to predict the response of their electrical conductivity to small uniaxial strains for unoriented d-UHMWPE-based composites with the extremely segregated structure made of MWCNTs was shown in previous works [38,39]. The numerical model considered the actual structure of a d-UHMWPE-based composite, which was produced using the solid-state compression of a dry mixture of the components. The composite structure was reconstructed numerically by processing images obtained using electron microscopy during the focused ion beam etching of the composite surface. The results of the numerical simulation enabled the formulation of conclusions regarding the multi-scale character of the structure of the solid-state processed composites.

In the study conducted by Zabolotnov et al. [40], the polymerization filling method was employed to fabricate nanocomposites comprising UHMWPE and ultralow graphene content (0.0065–0.019 vol.%). The strain–strength properties under the tensile stress of the composite samples were investigated as a function of graphene content. The results demonstrated that the tensile strength and relative elongation of the composites exhibited notable alterations in response to minor variations in graphene content, in comparison to unfilled UHMWPE. This study presents an illustrative example of a UHMWPE-based composite system with a structure that is diametrically opposed to the extreme segregation of the filler within the composite volume.

In the work of Shiyanova et al. [41], it was demonstrated that the physical properties of segregated network polymer composites are strongly dependent on the polymer matrix. Notably, the incorporation of reduced graphene oxide resulted in a 12-order-of-magnitude increase in electrical conductivity up to 0.7 S/m, accompanied by an increase in elastic modulus and a decrease in fracture strength in the poly(vinyl chloride)-based composites. At the same time, it was shown that the change in strength was insignificant for UHMWPE-based composites in comparison with unfilled polymers.

In the work of de Oliveira Aguiar et al. [42], nanocomposites were fabricated through the incorporation of thermally reduced and exfoliated graphene into UHMWPE, which was lubricated with mineral oil. The composites exhibited superior crystalline order and higher temperatures for the onset and maximum rate of thermal degradation. Dynamic mechanical analysis indicates enhanced viscoelastic stiffness from 1 up to 3 wt.% of the distinctive graphene filler incorporation, suggesting that this innovative graphene-based filler serves as reinforcement for UHMWPE-based nanocomposites. Similarly, in the work of Alaferdov et al. [43], highly conductive solid-state processed composites based on UHMWPE filled with graphite nanobelts were developed. The fabricated material exhibited excellent electrical conductivity (up to 40 S cm^−1^), high efficiency of electromagnetic interference shielding (near 35 dB for 100 μm thick samples), and good mechanical properties. These papers demonstrate that by carefully considering the type of filler for the solid-state processed UHWMPE-based composites it is possible to achieve a very versatile combination of the material properties, suitable for a specific practical application.

In the work of Drakopoulos et al. [29], composites comprising d-UHMWPE and gold nanoparticles were produced and oriented to achieve varying deformation ratios. The results of polarized light microscopy indicated that the gold nanoparticles were oriented in arrays that were aligned with the orientation of the polymer chains. Additionally, the change in optical absorbance in the visible spectrum indicated that the average size of gold aggregates increased with orientation. In another work of Drakopoulos et al. [44], it was demonstrated that the orientation of the composite material allows for a significant increase in its thermal conductivity in the direction of drawing. It was demonstrated that by selecting the optimal value of the orientation drawing ratio, it is possible to achieve the maximum ability of the material to accumulate and retain an electric charge. To comprehend the potential of such composites for a multitude of practical applications, further research is required on a broader range of filler types, their concentrations, and degrees of orientational deformation.

One of the main objectives of this study is to gain insight into the development of a composite material that exhibits both exceptional mechanical and functional characteristics. The novelty of our work is in a meticulous study of the processing stages of the composites, along with careful consideration of the type of d-UHMWPE RP and NPs, the NPs content, and orientation deformation degree. The segregated structure of the solid-state processed composites based on a d-UHMWPE RP is of particular interest due to its extreme character. The ability to controllably obtain composites with extremely segregated structures could provide excellent modeling opportunities for investigating the relationships between the filler type/structure, composite structure, and composite properties. Furthermore, given the significance of examining the response of the composite structure to external stimuli and the insights it can offer regarding the distribution of the filler and the filler mutual interaction, the study of the composite properties’ alterations during the UHMWPE-based composites orientation stage can be particularly beneficial. As evidenced by the literature review, a comprehensive study of the aspects of solid-state processing of the composites, based on nascent d-UHMWPE RP and modified with NPs of various types and content values, would be a necessary addition to the relevant research field.

The structure of the paper reflects the main objective of this study, which is to gather data on the aspects and specifics of the solid-state processing of the UHMWPE-based composites for every stage of the processing. In Section 2.1, the materials used in this work as the main components are described in detail, accompanied by supplementary data on the fillers and the d-UHMWPE RPs. Section 2.2 outlines the solid-state processing procedure used for obtaining oriented composite materials. In Section 2.3, the methods employed in this work to study the structure and properties of the resulting composite materials are presented. Section 3.1 addresses the specifics of the components mixing and the cold compaction of the resulting mixtures, in addition to the methodologies for enhancing the dispersion of the NPs within the composite volume. Section 3.2 presents the findings of the investigation into the structure and properties of the composites oriented to different deformation ratio values, along with the data on the piezoresistivity of the pre-deformed under homogeneous shear conditions composite samples. In Section 3.3, the impact of the uniaxial orientation deformation, which follows the homogeneous shear deformation, on the electrical conductivity of the UHMWPE-based composites, is analyzed. Section 3.4 presents the results of the thermal treatment effect on the electrical conductivity of both compacted and deformed under homogeneous shear conditions UHMWPE-based composite materials. Section 3.5 demonstrates the approach of combining the compaction and homogeneous shear deformation stages to obtain oriented UHMWPE-based composite tapes.

## 2. Materials and Methods

### 2.1. Materials

**Figure 1:**
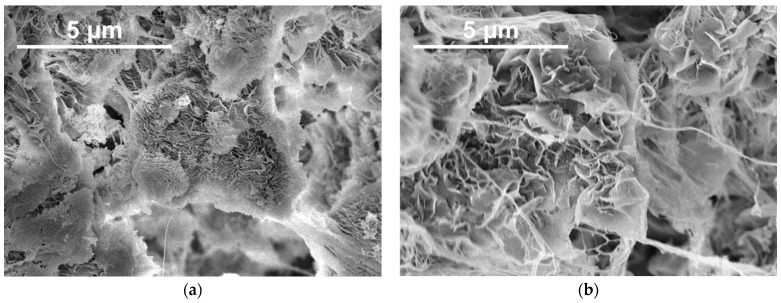
SEM images of the surfaces of the (**a**) UHMWPE-1 and (**b**) UHMWPE-2 nascent d-UHMWPE RP batches.

Multiple batches of nascent d-UHMWPE RP were used as polymer matrices in the investigated composites. The RPs were synthesized by Ivanchev et al. [33,45,46] and Gagieva et al. [47,48] and characterized by the average bulk density of 0.06 g/cm^3^. The d-UHMWPE RPs were selected based on the results of the research conducted by Ozerin et al. [33], where over 500 individual d-UHMWPE RP syntheses were tested. The selected d-UHMWPE RPs were previously successfully processed into materials with the highest tensile strength values among all the RPs studied. Depending on the synthesis parameters, the d-UHMWPE RP batches were designated in this work as “UHMWPE-x”, where “x” is the number of the batch. The selected scanning electron microscopy (SEM) images of the UHMWPE-1 and UHMWPE-2 RP batches are presented in Figure 1.

The following commercially available electroconductive carbon NPs of various types were selected as fillers: electrically conductive carbon black (CB) P267E (Omsk Carbon Black Plant, Omsk, Russia) [49]; graphite nanoplatelets (GNPs) AO-3 (Graphene Laboratories, Calverton, NY, USA); single-walled carbon nanotubes (SWCNTs) Tuball (OCSiAl, Luxembourg); MWCNTs NC3100 (“MWCNTs-1”, Nanocyl, Sambreville, Belgium); MWCNTs Graphistrength C100 (“MWCNTs-2”, Arkema, Colombes, France); MWCNTs Taunit M (“MWCNTs-3”, NanoTechCenter Ltd., Tambov, Russia). In addition, a special type of DWCNTs (INFRA Technology Ltd., Technological Institute for Superhard and Novel Carbon Materials, Troitsk, Russia) with exceptionally high length (up to several millimeters) and conductive properties was used. The full description of the DWCNTs used in this work can be found in the publication of Karaeva et al. [50]. The DWCNTs underwent preliminary chemical and thermal treatment, as detailed in the work of Filimonenkov et al. [51].

**Figure 2:**
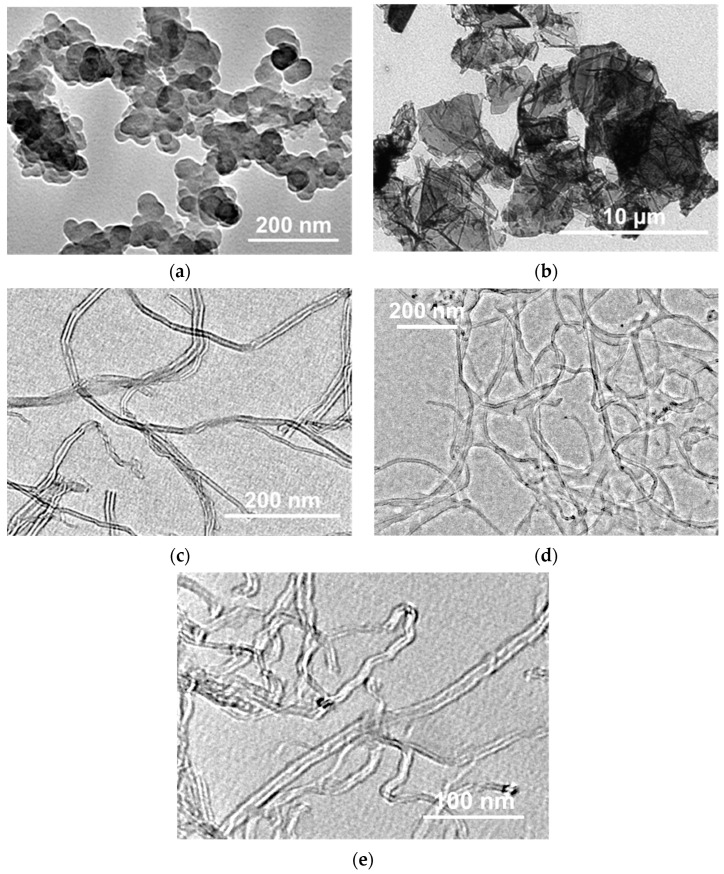
TEM images of the (**a**) CB, (**b**) GNP, (**c**) MWCNTs-1, (**d**) MWCNTs-2, and (**e**) MWCNTs-3 powders.

The transmission electron microscopy (TEM) images of the NP powders used in this work are presented in Figure 2. The detailed specification and TEM images of the SWCNTs can be found in the publication of Krestinin et al. [52].

**Figure 3:**
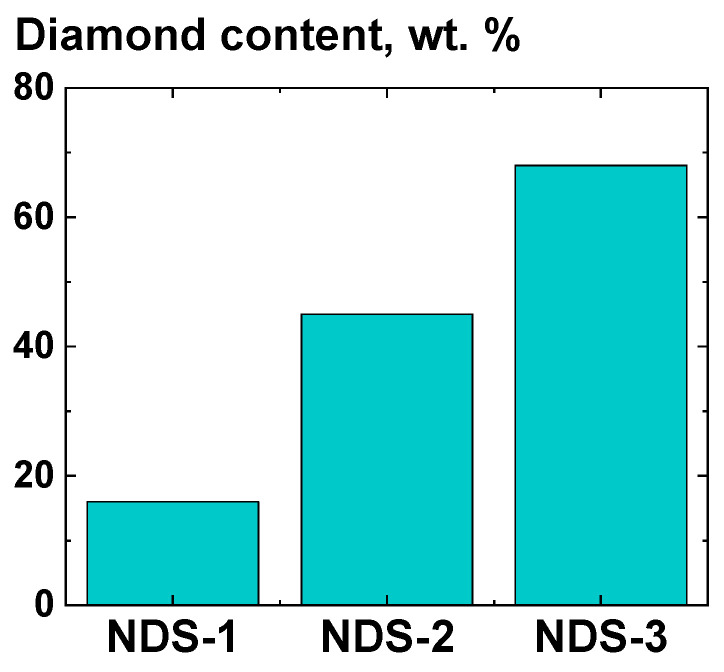
Diamond weight content values in the NDS powders of various types used in this work. The content values were calculated from the analysis of the X-ray diffractograms.

Three distinct types of nanodiamond soot (NDS), synthesized under varying conditions (Special Design and Technological Bureau “Technolog”, St. Petersburg, Russia), were used to provide additional information on the influence of the filler composition on the mechanical properties of UHMWPE-based composites. The NDS batches, designated as “NDS-1”, “NDS-2”, and “NDS-3” in this study, were obtained through the detonation of 2,4,6-trinitrotoluene (TNT), a 1:1 mass ratio mixture of TNT and 1,3,5-trinitro-1,3,5-triazinane, and 2,4,6-trinitrophenylmethylnitramine, respectively. Corresponding synthesis procedures were previously described in detail by Dolmatov et al. [53,54]. The results of a comprehensive investigation into the structure and properties of NDS-1 powder are presented in the work [55], where it was shown that the NDS-1 powder consisted of only two phases—diamond and graphite in the form of nanoflakes and nanoribbons. X-ray diffraction (XRD) analysis was conducted on all the selected NDS types to ascertain the weight fraction of the diamond phase present in the powder samples. The results of the analysis are shown in Figure 3.

### 2.2. Composites Preparation

**Figure 4:**
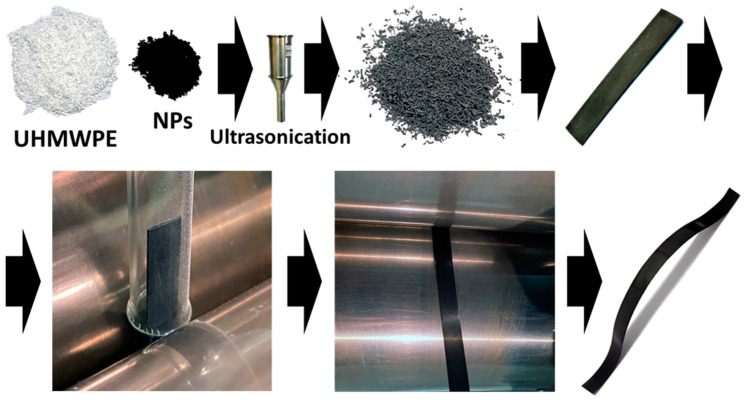
Scheme of preparation of the oriented tape samples of the composites based on d-UHMWPE RPs and various types of NPs.

The scheme of the procedure for the preparation of oriented d-UHMWPE-based composite samples is presented in Figure 4.

The sample preparation procedure comprised the preliminary ultrasonication of NPs in hexane, followed by the addition of 0.5 g of a selected d-UHMWPE RP into the NPs’ dispersion. The resulting mixture was subjected to an additional ultrasound treatment for a specified duration. After the ultrasonication, the mixtures were dried out at room temperature until complete hexane removal.

The ultrasound treatment of NP dispersions and the mixtures of a d-UHMWPE RP and NPs was conducted using an I100-840 ultrasonic disperser (LLC «Ultrasonic technique—INLAB», Saint-Petersburg, Russia), characterized by the frequency of 22.5 kHz, adjustable power output (up to 1 kW), and running water cooling system. The ultrasound treatment at 75% of the maximal power output of the dispenser yielded the most reproducible results for the conductivity of the obtained compacted composite samples. The ultrasound treatment was conducted by submerging the ultrasonic probe into a vessel of 100 mL filled with 50 mL of hexane. To prevent overheating of the mixtures, the vessel was placed inside an ice water bath.

In order to study the effect of the preliminary ultrasound treatment of NPs in hexane on the characteristics of the composite materials, 1.5 wt.% of MWCNTs-3 was used as the composites’ filling. The MWCNTs-3 content value of 1.5 wt.% was situated in close proximity to the percolation threshold, as determined through a preliminary screening of the percolation threshold for the solid-state processing method. This ensured that the resistivity of the composite samples with the 1.5 wt.% of MWCNTs to be especially sensitive to variation in the processing parameters. The standard duration of the ultrasound treatment of the mixture following the addition of d-UHMWPE RP to hexane was 10 min. In order to minimize the agglomeration and sedimentation of the filler in hexane prior to the introduction of the d-UHMWPE RP into the liquid medium, the RP was added into hexane directly during the ultrasound treatment of the NPs.

The obtained dry mixtures of UHMWPE and the NPs were molded (compacted) into rectangular samples with the dimensions of 0.05 × 10 × 1 cm. The molding was conducted in a closed mold at room temperature under a pressure of 300 MPa. In accordance with the findings of Aulov et al. [56], the value of 300 MPa for the compaction pressure was sufficient to ensure low porosity and high tensile strength of the compacted samples.

The obtained composite samples were subjected to deformation under homogeneous shear conditions by rolling (calendaring) them between two steel rollers with a diameter of 155 mm. The rollers were heated up to the temperature of 120 °C and rotated at the speed of 400 mm/min. The clearance distance between the rollers determined the ratio of deformation under homogeneous shear conditions [32,57]. The deformation ratio was evaluated from the change in the length of the samples.

The resulting monolithic oriented composite tapes were subjected to further uniaxial orientation drawing using the SpinLine processing equipment (DACA Instruments, Goleta, CA, USA). During this process, the tapes were in contact with a metal pin heated to 139 °C.

### 2.3. Methods of Investigation

Transmission electron microscopy (TEM) studies of the filler powder samples were conducted using a fully digital 200 kV scanning transmission electron microscope (S/TEM) FEI Tecnai Osiris system (FEI Company, Hillsboro, OR, USA). The samples were prepared by depositing the NP solutions onto a holey carbon film placed onto a golden grid.

SEM studies of the d-UHMWPE RPs, mixtures of the RPs with various NPs, and the cleavages of the compacted and oriented composite samples were conducted using a Supra 50 VP LEO scanning electron microscope (Carl Zeiss AG, Oberkochen, Germany) with an INCA Energy + Oxford system for microanalysis. Additionally, a Regulus SU8000 (Hitachi, Ibaraki, Japan) scanning electron microscope was used to obtain supplementary SEM data on the composite samples. The cleavages of the composite specimens were obtained via freeze–fracturing at the temperature of liquid nitrogen. SEM studies of the surfaces of the oriented composite tapes were conducted using a JSM-6000 (Neoscope II) scanning electron microscope (Jeol, Tokyo, Japan).

XRD diffractograms (2θ = 10–105°) were obtained using a D8 Advance (Bruker, Billerica, MA, USA) powder diffractometer (λ(CuK_α_) = 1.5418 Å) equipped with a focusing germanium crystal monochromator, a Ni-filter, and a LYNXEYE 1D scintillating detector. XRD diffractograms were recorded in the transmission mode. The filler powder samples were placed in between two layers of an amorphous poly(ethylene terephthalate) film. The processing of diffraction patterns (peak separation, corrections for non-coherent background scattering, and scattering from poly(ethylene terephalate)) was performed using the OriginPro 2018b software package. The wide-angle XRD patterns were registered using a NANOSTAR (Bruker, Billerica, MA, USA) device equipped with a two-dimensional detector of the CuK_α_ radiation.

Direct current electrical conductivity of the filler powders was measured using a 34,401A multimeter (Agilent, Santa Clara, CA, USA) and the four-probe method.

**Figure 5:**
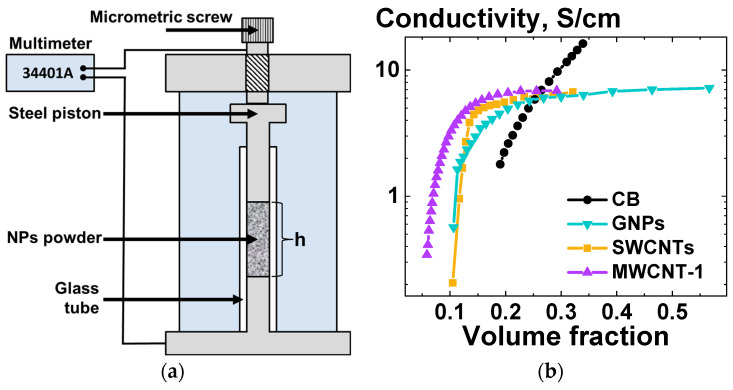
(**a**) Scheme of the appliance used for the measurements of the electrical conductivity of the carbon-based filler powders; (**b**) Electrical conductivity of the NP powders of various types versus volume fraction of the NPs in the measurement cell.

To be able to evaluate the potential of the selected carbon NPs as possible fillers for the improvement of electrical conductivity of the UHMWPE-based materials, the electrical conductivity of the filler powders was measured depending on the NPs volume fraction in the measurement cell. A special appliance was used for the variation of the filler powder volume fraction while simultaneously allowing us to measure the electrical resistance of the powder. The scheme of the appliance used and the results of the NP powders’ electrical conductivity measurements are presented in Figure 5a. For calculation of the carbon nanofillers volume fraction values, the density value of 2.2 g/m^3^ was used [58].

As can be seen in Figure 5b, for all filler powder samples, except CB, the rate of increase in conductivity with increasing filler volume fraction becomes significantly lower after a certain volume fraction value is reached. The relatively low overall measured values of electrical conductivity of the highly compacted GNPs and CNTs and powders can be attributed to the anisotropy of the properties of such NPs. As the NP powders were compacted uniaxially in order to change their bulk density (Figure 5a), this resulted in a preferential planar orientation of the GNP particles in the plane perpendicular to the conductivity measurement direction. For GNPs, the in-plane electrical conductivity is >2 orders of magnitude higher than the out-of-plane conductivity [59]. In the case of CNTs, the orientation of the NPs in the plane perpendicular to the direction of the conductivity measurement leads to a significant increase in the number of resistive contacts in the measurement direction. This also results in unexpectedly low measured electrical conductivity values. The conductivity of both GNPs and MWCNTs at high-volume fractions was found to be consistent with the previously reported results for the same types of NPs [59]. In the case of CB particles, the increase in electrical conductivity was found to be linear with increasing CB volume fraction. The values of CB conductivity at high-volume fractions were notably higher than those observed for CNT and GNP powders. Conversely, the CB powder exhibited the lowest conductivity among all the NP powders investigated, as reported in the literature [49,59]. This suggests that the alignment of anisotropic fillers may play a pivotal role in the electrophysical characteristics of the resulting composites.

The temperature dependence of the electrical conductivity of the compacted samples was studied in a specially designed measuring cell with a liquid nitrogen cooling system, with a minimum recorded temperature of 110 K. Copper wires were attached to the composite sample surfaces with an electrically conductive adhesive and then connected to the ports leading out of the cell. The temperature was increased at a rate of 2 K/min, with a 5-min pause every 10 K for electrical resistance measurements.

The annealing of the oriented composite samples was conducted in an EVCLIM-DVO-52 (Erstevak Ltd., Moscow, Russia) vacuum oven. The samples were annealed for one hour, after which they were allowed to cool to room temperature. Thereafter, the electrical conductivity of the cooled samples was measured.

The universal testing machine Ez Test Ez-LX (Shimadzu, Kyoto, Japan) equipped with a load cell of 500 N was used for the mechanical testing of the UHMWPE-based samples. The traverse speed was set to 2 mm/min until a pretension of 0.5 N was reached. Thereafter, the strain rate was set to 1 mm/min with the initial distance between the clamps set at 50 mm. For each specimen, three repeatable curves were obtained.

## 3. Results and Discussion

As the initial step of the work, the methodology used for the components mixing and the subsequent cold compaction of the resulting mixtures was elucidated (Section 3.1). Furthermore, potential ways to optimize the processing parameters to achieve the most effective dispersion of the fillers were explored.

The next step of the work (Section 3.2) was the study of the aspects of homogeneous shear deformation of the composite samples. The structure and properties of the composites oriented to different deformation ratio values were studied. This allowed us to understand the influence of the presence of the filler within the composite volume on the orientational strengthening of the composites. The piezoresistivity of the pre-deformed under homogeneous shear conditions composite samples was studied.

In the next step, the structure and properties of the UHMWPE-based composites modified with a special type of DWCNTs, characterized by the exceptional length, were investigated (Section 3.3). The impact of the uniaxial orientation deformation, which followed the homogeneous shear deformation, on the electrical conductivity of the UHMWPE-based composites, was analyzed. The potential for obtaining highly strong and electrically conductive tapes was assessed. The capability of the SEM method for examining the structure of the highly oriented UHMWPE-based composite tapes was demonstrated.

The next stage of the research (Section 3.4) involved an investigation into the temperature dependencies of the electrical conductivity of both compacted and deformed under homogeneous shear conditions UHMWPE-based composite materials. The impact of the structural levels of the solid-state processed composites on the composite electrical conductivity was studied, along with the specifics of the composite structural changes occurring during the material transition into the melted state.

Finally, the possibility of combining the compaction and homogeneous shear deformation stages to obtain oriented UHMWPE-based composite tapes was demonstrated for the mixtures of d-UHMWPE RP and NPs of various types (Section 3.5).

### 3.1. Mixing and Compaction

#### 3.1.1. Mixtures of the d-UHMWPE RP and NPs

As previously stated in Section 1 and Section 2.2, the solid-state processing approach for obtaining UHMWPE-based composites requires the stage of covering the d-UHMWPE RP particles with the filler NPs. This occurs during the ultrasound treatment of the mixture in the liquid medium (hexane), where the filler NPs are mechanically attached to the RP irregularities, including pores and protrusions. The processing parameters, such as the duration of preliminary ultrasonication of NPs in hexane, or the duration of ultrasonication of the UHMWPE and NPs mixtures, play a critical role in the efficiency of the NPs coverage of the RP particles. These parameters also determine the number of NPs remaining in the liquid medium at the end of the ultrasound treatment. As the primary objective of this study, the ways to optimize these processing parameters were studied.

**Figure 6:**
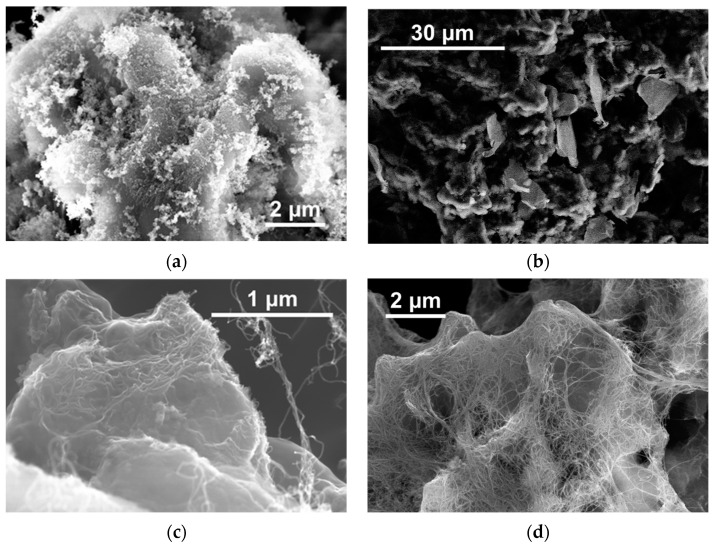
SEM images of the surfaces of the UHMWPE-1 RP after its ultrasound treatment in hexane in the presence of (**a**) 10 wt.% CB, (**b**) 10 wt.% GNPs, (**c**) 2 wt.% SWCNTs, or (**d**) 2 wt.% MWCNTs-1.

The NPs distribution on the surface of the d-UHMWPE RP particles was controlled using SEM. Figure 6 shows the SEM images of the surfaces of UHMWPE-1 RP particles following their ultrasonication in hexane for 10 min in the presence of 10 wt.% of CB (Figure 6a), 10 wt.% of GNPs (Figure 6b), 2 wt.% of SWCNTs (Figure 6c), or 2 wt.% of MWCNTs-1 (Figure 6d).

It can be observed that the surfaces of the UHMWPE-1 RP particles were coated by the NPs in the obtained mixtures, exhibiting varying degrees of uniformity dependent on the type of filler. Some types of MWCNTs (Figure 6c,d) formed agglomerates on the d-UHMWPE RP particle surfaces with sizes in the range of 200−400 nm. Conversely, in some areas, the RP particles demonstrated minimal to no coating by the filler.

It was determined that the use of ultrasonication for the preparation of the d-UHMWPE RP and NPs mixtures is effective only up to a certain NP content value. This is due to the inability of the RP particles to efficiently capture the NPs onto their surface at higher NP concentrations. Furthermore, for such high-aspect ratio particles, such as CNTs, at exceedingly high content values (>15 wt.%), the d-UHMWPE RP and the NPs mixtures began to exhibit markedly increased viscosity. This was due to the formation of a gel-like CNTs network in hexane in the presence of the d-UHMWPE RP, which further diminished the efficiency of the NP coverage of the RP particles.

A direct investigation of the distribution of the NPs on the surface of the d-UHMWPE RPs using the SEM method for each set of processing parameters would be impractical. Therefore, the quality of the NPs distribution was controlled by measuring the electrical conductivity of the compacted composite samples.

#### 3.1.2. Compacted Composite Samples

**Figure 7:**
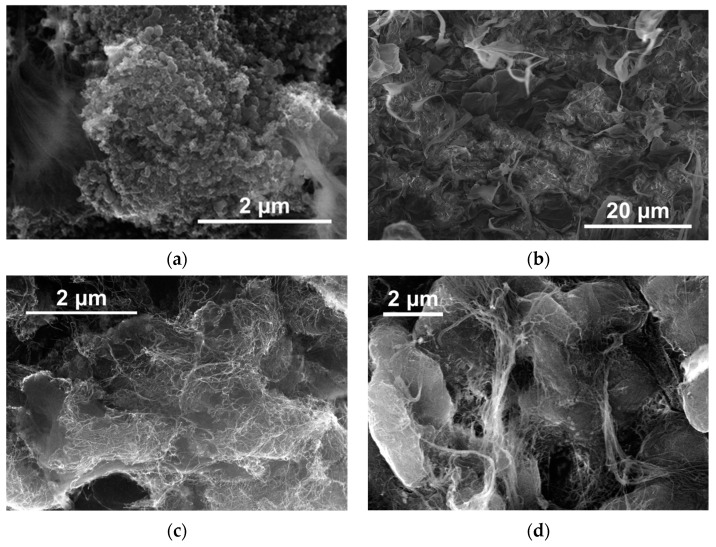
SEM images of the surfaces of the low-temperature cleavages of the compacted mixtures of UHMWPE-1 RP and (**a**) 10 wt.% CB, (**b**) 10 wt.% GNPs, (**c**) 2 wt.% SWCNTs, or (**d**) 2 wt.% MWCNTs-1.

Figure 7 depicts the selected SEM images for the cleavages of compacted composite samples comprising UHMWPE-1 and 10 wt.% of CB (Figure 7a), 10 wt.% of GNPs (Figure 7b), 2 wt.% of SWCNTs (Figure 7c), or 2wt.% of MWCNTs-1 (Figure 7d).

As anticipated, the solid-state (below the melting point of the selected UHMWPE, ~141.5 ^°^C [60]) processing method of the UHMWPE-based composites resulted in the extreme segregation of the filler NPs within the composite volume. This phenomenon is evident in all of the obtained SEM images of the compacted composite samples (Figure 7), wherein the localization of the NPs on the boundaries of the UHMWPE grains is discernible. All of the studied composite samples exhibited fracturing along the boundaries of the compacted polymer particles during the preparation of samples for SEM investigations. This demonstrates the tenuous nature of the bonding between the polymer and the filler NPs.

#### 3.1.3. Effect of the d-UHMWPE RP Type and Preliminary Ultrasound Treatment of NPs

As the next step of the work, a variety of batches of d-UHMWPE RP was used to obtain the solid-state processed composites. As was mentioned in Section 2.1, the selected batches of the RP batches were selected based on their ability to be processed into a highly oriented state, which is characterized by exceptionally high modulus and tensile strength values.

**Figure 8:**
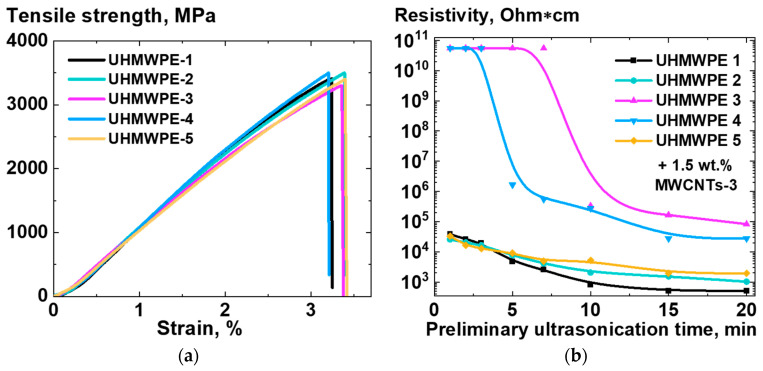
(**a**) The results of the mechanical testing of the maximally oriented UHMWPE tapes, obtained from five different batches of the d-UHMWPE RPs; (**b**) The specific resistivity of the compacted composite samples based on the same set of the d-UHMWPE RP batches, modified with 1.5 wt.% of MWCNTs-3, as a function of the duration of the NP powder preliminary ultrasound treatment in hexane prior to the addition of the UHMWPE into the NP dispersion.

The results of the mechanical testing of the highly oriented (total deformation ratio ~ 130) tapes, obtained from the selected RP batches using the solid-state processing procedure described in Section 2.2, are presented in Figure 8a. Additionally, the effect of preliminary ultrasonication of the NP powder in hexane on the electrical resistivity of the resulting compacted composite samples was investigated for the selected d-UHMWPE RPs. The results of the study are presented in Figure 8b. The variation of the preliminary ultrasound treatment duration allowed us to alter the initial dispersion of the NPs at the moment of the addition of the d-UHMWPE RP into the liquid medium. This influenced the efficacy of how the NP aggregates were captured onto the RP particles’ surface, or their capacity to enter the pores of the RP particles.

As anticipated, all selected d-UHMWPE RPs were successfully processed into highly oriented tapes with tensile strength values of approximately 3.35 ± 0.05 GPa (Figure 8a). As previously stated, this result was due to the chosen types of d-UHMWPE RPs being characterized by the necessary structure parameters for effective solid-state processing [28,32,33,35]. At the same time, not all selected RPs allowed the formation of highly conductive NP structures on the surfaces of the RP particles. No noticeable electrical conductivity was observed for the mixtures of a number of RP batches with the MWCNTs after the compaction, independent of the duration of preliminary ultrasonication or ultrasonication of the mixtures. The data for such RPs were not included in Figure 8. This important result indicates that the structural parameters of the RPs required for the effective distribution of the NPs at the surface of the RP particles may differ from those necessary for the polymer to achieve a highly oriented, high-strength state. Particularly, this could be due to the scale of the UHMWPE crystalline lamellas being significantly lower than the characteristic size scale of the NP clusters [33].

In general, preliminary ultrasound treatment of fillers such as MWCNTs for as little as five minutes was observed to result in a reduction in resistivity up to five times for the most promising batches of d-UHMWPE RP (Figure 8b). For the subsequent investigations, d-UHMWPE RP UHMWPE-1, which demonstrated the best processibility into highly electrically conductive materials, was selected as the matrix for the composites.

It was observed that not all the selected types of NPs responded equally to the preliminary ultrasound treatment. For instance, the DWCNTs formed large agglomerates after ~2 min of ultrasound treatment prior to the addition of the d-UHMWPE RP into hexane. Consequently, in order to standardize the composite preparation procedure, the stage of preliminary treatment of the NPs was omitted.

#### 3.1.4. Duration of Ultrasonication of the d-UHMWPE RP and NPs Mixtures

**Figure 9:**
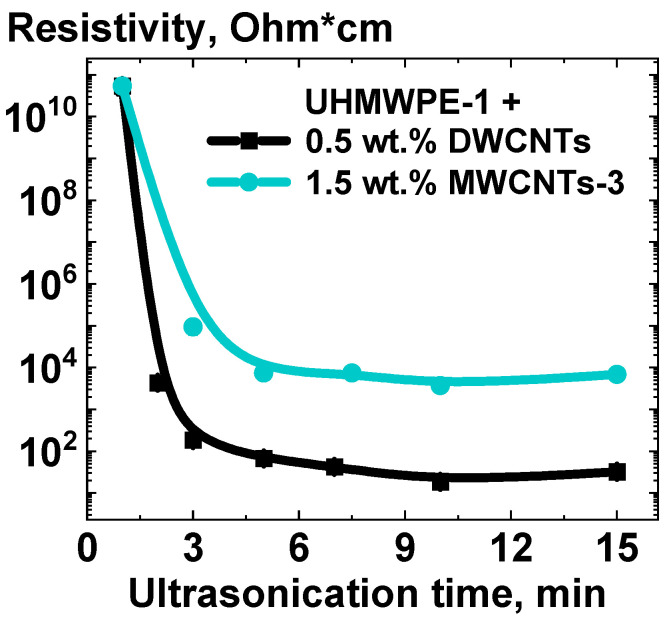
Specific resistivity of the compacted mixtures of UHMWPE-1 RP and 0.5 wt.% DWCNTs or 1.5 wt.% of MWCNTs-3 versus the duration of the ultrasound treatment of the mixtures of the d-UHMWPE RP and the filler powder in hexane.

The next step was to study the resistivity of the composite materials based on UHMWPE-1 RP, depending on the time of the ultrasound treatment of the RPs together with the NPs in hexane. The results of the resistivity at the time of ultrasonication of the NPs together with UHMWPE-1 RP in hexane are presented in Figure 9.

The lowest electrical resistivity of the composite samples was observed after 10 min of ultrasound treatment of the RP and NPs mixtures for both the studied compositions. For composites filled with MWCNTs, no significant changes in the electrical resistivity were noted after 10 min. In contrast, for composites filled with DWCNTs, a slight increase in resistivity was recorded. This was attributed to both the DWCNTs’ microgel formation and the mechanical breakdown of the DWCNTs into shorter fragments.

In order to standardize the composite preparation process, a 10-min ultrasound treatment duration was selected for the UHMPWE RP and NPs mixtures in hexane.

#### 3.1.5. Electrical Conductivity Dependences on the NP Content

**Figure 10:**
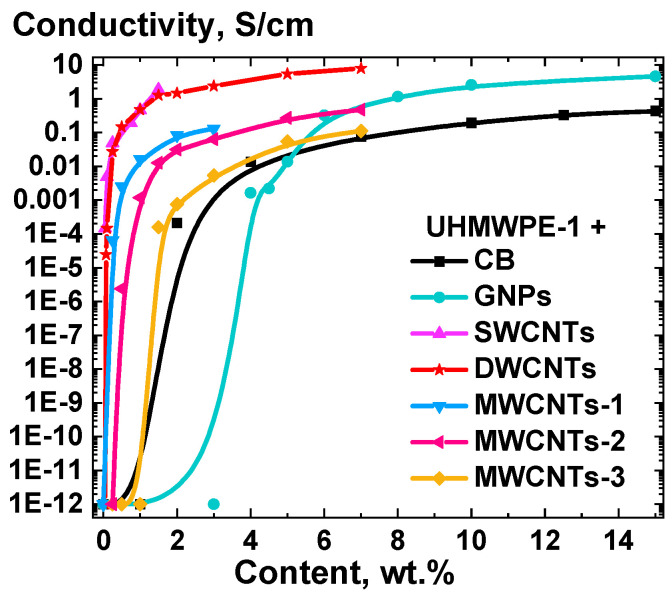
Electrical conductivity of the compacted composite samples based on UHMWPE-1 modified with CB, GNPs, SWCNTs, DWCNTs, MWCNTs-1, MWCNTs-2, and MWCNTs-3, as a function of the filler weight content in the composite samples.

The electrical conductivity of composite materials, based on UHMWPE-1 RP and obtained under standardized conditions of the solid-state processing, was investigated for the selected types of NPs depending on the degree of the filling of the composites. The results of the measurements are presented in Figure 10.

The percolation threshold values for all the NP types were estimated by approximating the electrical conductivity dependencies on the degree of filling with a sigmoidal Boltzmann function [61]. As anticipated based on percolation theory [62], the lowest percolation threshold (~0.05 vol.%) was identified for the SWCNTs as the filler, with the value of ~0.03 vol.% obtained for composites containing the DWCNTs. The highest values were obtained for GNPs (~2.5 vol.%) and CB (~1.6 vol.%) as the fillers. The observed percolation threshold values are notably lower than the values predicted by percolation theory for a uniform distribution of filers with similar aspect ratios [63,64]. This discrepancy can be attributed to the formation of a segregated conductive structure from a three-dimensionally distributed filler located solely on the boundaries of the d-UHMWPE RP particles.

The percolation threshold values presented in Figure 10 should be considered as upper estimates. Particularly, these values were obtained for composites that were not processed optimally, e.g., without the optimized preliminary ultrasonication stage. The latter results in a notable reduction in the resistivity of the composites (Figure 8), while also affecting the quality of the NPs dispersion on the surface of the d-UHMWPE RP particles (Figure 6). It is anticipated that the percolation threshold values will be considerably lower for processing parameters that are optimized for each type of filler.

### 3.2. Homogeneous Shear Deformation and Uniaxial Orientation

The next stage of the work involved a study of the structure and properties of oriented through deformation under homogeneous shear conditions UHMWPE-based composites.

#### 3.2.1. SEM of the Oriented Composite Samples

**Figure 11:**
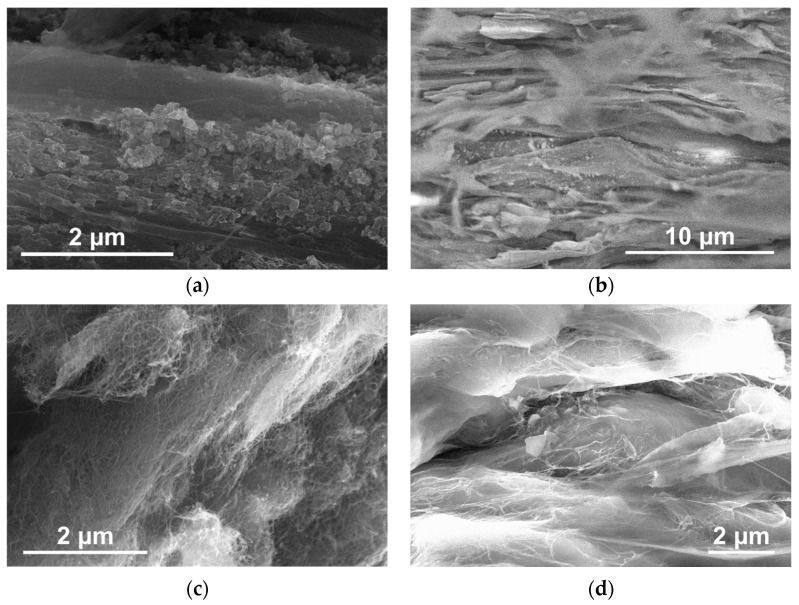
SEM images of the surfaces of the low-temperature cleavages of the deformed to the ratio value of ~6 composite tape samples based on UHMWPE-1 filled with (**a**) 10 wt.% CB, (**b**) 10 wt.% GNPs, (**c**) 2 wt.% SWCNTs, and (**d**) 2 wt.% MWCNTs-1.

The compacted and oriented composite samples, based on UHMWPE-1 RP and various NPs, were examined using SEM. Figure 11 presents the selected results of the SEM investigation for the cleavages of UHMWPE-based composite samples with a deformation ratio of ~6. The samples contained 10 wt.% of CB (Figure 11a), 10 wt.% of GNPs (Figure 11b), 2 wt.% of SWCNTs (Figure 11c), and 2 wt.% of MWCNTs-1 (Figure 11d).

It is notable that even for a relatively high value of deformation ratio (~6), the distribution of the NPs can be considered more or less uniform for any studied type of the NPs (Figure 11). Concurrently, the composite structure remains highly segregated, with the filler layer following the elongated UHMWPE grain boundaries. For the anisotropic NPs, such as GNPs (Figure 11b), MWCNTs (Figure 11c), or SWCNTs (Figure 11d), the orientation of the polymer matrix is accompanied by the orientation of the filler particles as well.

#### 3.2.2. Mechanical and Electrical Properties of the Oriented Composites

**Figure 12:**
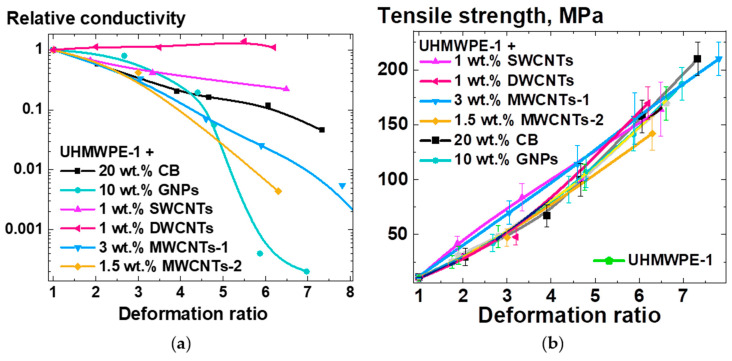
Dependencies of (**a**) relative conductivity and (**b**) tensile strength on the homogeneous shear deformation ratio for the samples of composites based on UHMWPE-1 filled with 20 wt.% of CB, 10 wt.% of GNPs, 1 wt.% of SWCNTs, 1 wt.% of DWCNTs, 3 wt.% of MWCNTs-1, or 1.5 wt.% of MWCNTs-2. The relative conductivity was calculated by dividing the conductivity value for the specific deformation ratio by the conductivity value, corresponding to the nondeformed (deformation ratio 1) composite sample of the same composition.

The mechanical characteristics and electrical conductivity were examined for the compacted and oriented under homogeneous shear conditions samples of composites based on UHMWPE-1 RP and the selected types of NPs. Figure 12 shows the dependencies of the relative conductivity (Figure 12a) and tensile strength (Figure 12b) on the deformation ratio for the samples of UHMWPE-based composite samples containing various NPs. The relative conductivity was calculated by dividing the sample conductivity value by the conductivity value for the corresponding non-deformed sample.

The majority of oriented UHMWPE-based samples exhibited a decrease in conductivity with an increase in deformation ratio (Figure 12a). The composites filled with GNPs demonstrated a threshold in their conductivity dependence on the deformation ratio. In contrast, the exceptionally long DWCNTs used in this study [50] showed no decrease in conductivity for filler concentrations above 0.5 wt.% following deformation under homogeneous shear conditions up to a deformation ratio value of ~6. This unique result demonstrates a significant influence of the aspect ratio of the filler particles on the deformation response of the electrophysical characteristics of the solid-state processed UHMWPE-based composites.

The results of the mechanical testing of the UHMWPE-based composite samples demonstrated that the UHMWPE matrix reinforcement through orientation was equally effective for the filler-free UHMWPE and for the UHMWPE-based composites filled with the carbon NPs of all the studied types (Figure 12b). The tensile strength of the composites exhibited a nearly linear and almost identical change in relation to the deformational behavior of the non-filled UHMWPE.

**Figure 13:**
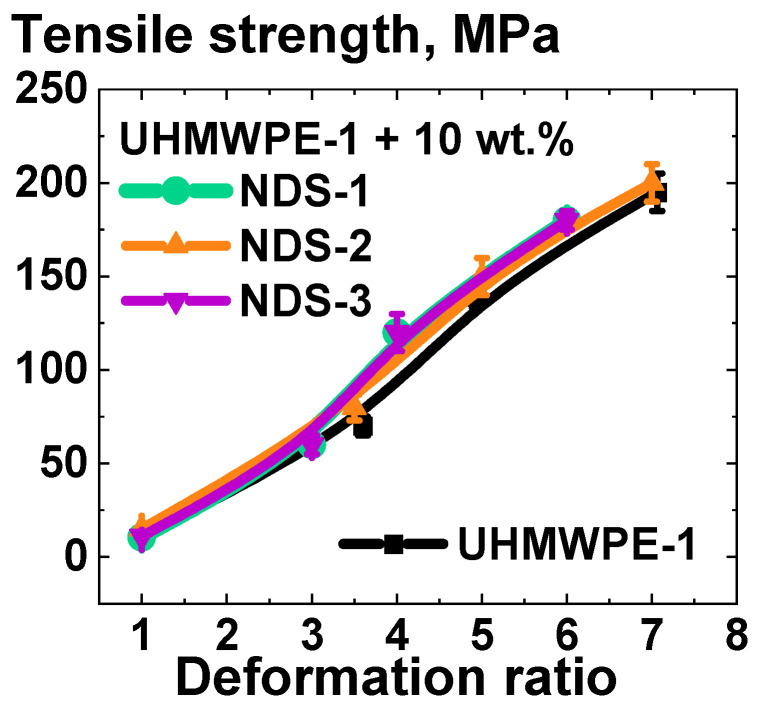
Dependence of tensile strength on homogeneous shear deformation ratio for the composites based on UHMWPE-1 with and without the addition of 10 wt.% of NDS of various types.

To further demonstrate the significant finding that the filling has a minimal impact on the mechanical properties of UHMWPE-based composites processed in the solid state, a unique set of NDS powders was employed for the modification of UHMWPE (see Section 2.1). The results of the mechanical testing of the UHMWPE-1-based composites with and without the addition of 10 wt.% NDS of various types are presented in Figure 13.

It was determined that the tensile strength values for the UHMWPE-based composites filled with the NDS, which exhibit a significantly disparate range of graphite/diamond content ratios (Figure 3), were found to be within the margin of error from one another and the tensile strength of the non-filled UHMWPE, irrespective of the deformation ratio (Figure 13).

Since it is not possible to fully cover the RP particle surface with NPs, with the scale of the crystalline lamellas (Figure 1, [33]) being significantly lower than the characteristic sizes of the NP clusters (Figure 2), it can be assumed that the necessary number of contacts between the UHMWPE crystalline lamellas are formed, and that solid-state processing is conducted independently of the filler type and its content.

#### 3.2.3. XRD Study of the Structure of the Oriented Composites

**Figure 14:**
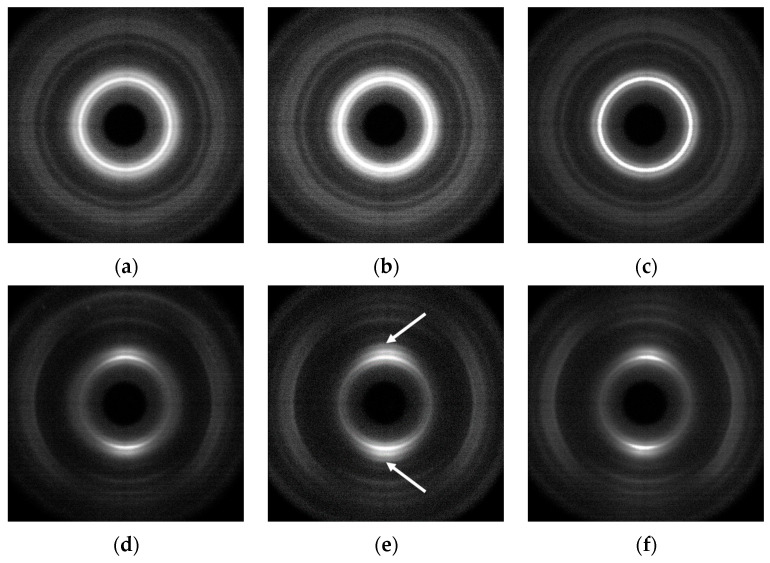
X-ray scattering patterns for the (**a**–**c**) compacted and (**d**–**f**) deformed under homogeneous shear conditions to the deformation ratio of ~4 composite tapes based on UHMWPE-1 mixed with (**a**,**d**) 20 wt.% CB, (**b**,**e**) 10 wt.% GNPs, or (**c**,**f**) 3 wt.% MWCNTs-1. The direction of the primary X-ray beam was perpendicular to the flat surface of the tapes. The orientation direction of the samples was horizontal. The graphite 002 reflection peak position for the composite samples filled with GNPs is indicated with the arrows in the X-ray scattering patterns (**b**,**e**).

To gain a deeper understanding of the effect of filler content on the mechanical properties of UHMWPE-based composites, wide-angle XRD studies were conducted on compacted and deformed samples. The resulting XRD patterns for the UHMWPE-1-based composites are shown in Figure 14.

The analysis of the ratios of the reflections corresponding to the crystalline phase of the UHMWPE in the longitudinal and transverse directions revealed that the structure of the UHMWPE was not significantly affected by the presence of any type of filler in the composite volume during the orientation strengthening (Figure 14). It was noted that the filler also exhibited a consistent orientation during the deformation process. The ratio of reflections in the longitudinal and transverse directions for the filler changed in proportion to the changes in reflections of the polymer phase. This result is particularly noticeable for anisotropic fillers such as GNPs (Figure 14b,e).

The obtained results demonstrate that the UHMWPE does not perceive the presence of the filler in any composition during solid-state processing and orientation strengthening. This phenomenon may be attributed to the highly segregated structure of the studied polymer composites.

#### 3.2.4. Piezoresistivity of the Oriented Composites

**Figure 15:**
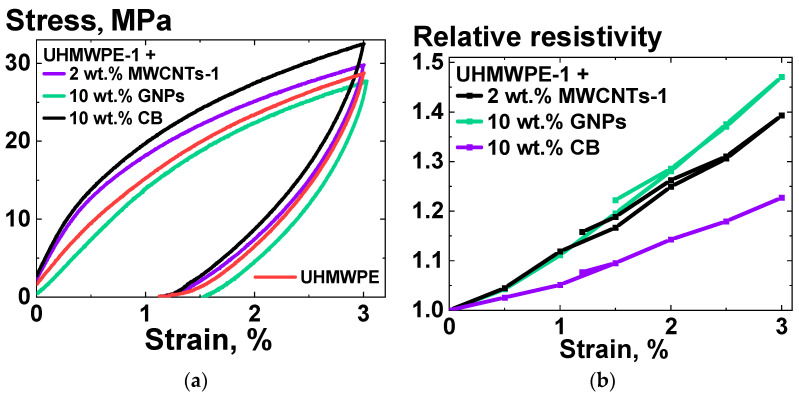
Dependencies of (**a**) stress and (**b**) relative resistivity on the strain value during the tensile deformation of the preliminary deformed under the homogeneous shear conditions to the deformation ratio value of ~5 composite samples based on UHMWPE-1 filled with 10 wt.% of CB, 10 wt.% of GNPs, or 2 wt.% of MWCNTs-1. Relative resistivity was calculated through the division of the resistivity value for the specified strain using the resistivity value, corresponding to the unloaded (strain 0) state.

The resistivity response to the uniaxial deformation under normal conditions was studied for the UHMWPE-based composite samples, deformed under homogeneous shear conditions, depending on the filler type. The deformation curves for the composites based on UHMWPE-1 and the dependencies of the relative resistivity of the composites on the uniaxial strain are shown in Figure 15.

The relative change in resistivity for all the composites under investigation was almost linear and reversible as long as the deformation was reversible (Figure 15). The rate of increase in resistivity due to the uniaxial deformation (Figure 15b) was significantly higher than that observed under homogeneous shear conditions (Figure 12b). The proportionality coefficients between the relative resistivity increase and strain for composites filled with different types of fillers did not align with the corresponding differences in the stress–strain dependencies. These results reinforce the findings regarding the independence of the deformational behavior of the polymer matrix and the electrically conductive segregated network within the composite volume.

### 3.3. Composites Filled with the DWCNTs

The weak dependency of the relative conductivity on the ratio of deformation under homogeneous shear conditions (Figure 12a) demonstrated the uniqueness of the samples of composites based on UHMWPE-1 filled with DWCNTs. This type of filler allowed the production of strengthened oriented composite materials without any noticeable changes in the electrical conductivity, making it an ideal choice for further investigation into the solid-state processing of UHMWPE-based composites.

#### 3.3.1. Uniaxial Deformation of the Oriented Samples

One of the most important stages in the solid-state processing procedure is the uniaxial orientation deformation stage (see Section 2.2), which follows the homogeneous shear deformation stage [32,33]. This stage results in the formation of a highly oriented state of the UHMWPE-based materials, which exhibits tensile strength > 3 GPa (Figure 8a) and elastic modulus > 120 GPa. The maximum overall deformation ratio for the samples processed from the most promising d-UHMWPE RPs can exceed 130, comprising homogeneous shear deformation and subsequent uniaxial deformation [32]. Given that the uniaxial orientation deformation is conducted at a temperature proximate to the melting point of the UHMWPE (see Section 2.2), it is reasonable to hypothesize that the response of the electrical conductivity of the UHMWPE-based composites will differ from the response of the composite conductivity during tensile loading at room temperature (Figure 15b).

**Figure 16:**
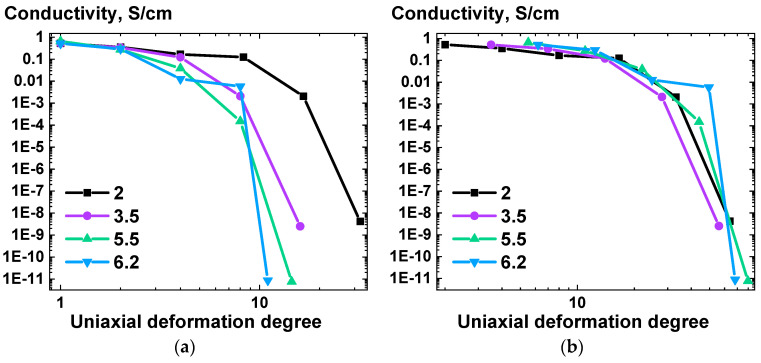
Dependencies of conductivity on the degree of the uniaxial deformation for the deformed to the different homogeneous sheer deformation ratio values (2, 3.5, 5.5, 6.2) composite samples based on UHMWPE-1 filled with 1 wt.% of DWCNTs. In the graph (**a**), for each sample, the initial value of the uniaxial deformation degree was set as “1” in the corresponding curves. In the graph (**b**), for each sample, the initial uniaxial deformation values were set to be equal to the sample homogeneous shear deformation ratio value in the corresponding curves.

In order to investigate the impact of uniaxial deformation on the conductivity of UHMWPE-based composites, the samples were subjected to preliminary deformation under homogeneous shear conditions with varying deformation ratios (2, 3.5, 5.5, 6.2). Subsequently, the samples were subjected to additional uniaxial deformation at 139 °C to varying degrees. The results of the conductivity measurements for the obtained highly oriented composite tapes are presented in Figure 16.

As can be seen in Figure 16a, at specific deformation degree values, the conductivity of the DWCTNs-filled samples showed a decline. The uniaxial deformation degree value at which a notable loss of conductivity was observed was dependent upon the value of the preliminary deformation ratio.

To ascertain the influence of each deformation stage on the conductivity changes, the initial uniaxial deformation values were adjusted to align with the corresponding homogeneous shear deformation ratio values of the samples, as illustrated in Figure 16b. This allowed for an important observation to be made: the rate of conductivity decline is independent of the initial deformation under homogeneous shear conditions ratio, provided that the overall deformation ratio (homogeneous shear deformation followed by uniaxial deformation) is taken into account.

#### 3.3.2. Mechanical Properties of the Highly Oriented Composite Tapes

**Figure 17:**
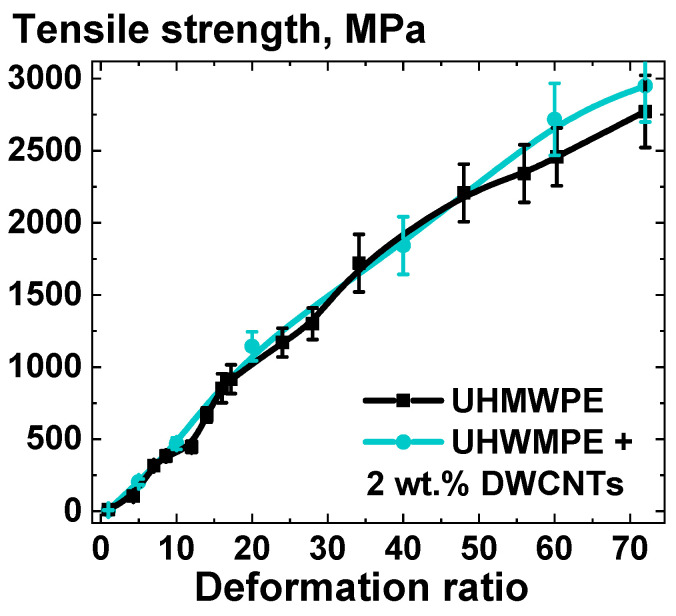
Dependence of tensile strength on the combined deformation ratio (homogeneous shear deformation followed by the uniaxial deformation) for the oriented tapes based on UHMWPE-1 with and without the addition of 2 wt.% of DWCNTs.

To investigate the potential capabilities of the composites based on solid-state processed UHMWPE, the mechanical characteristics of the highly oriented tapes of the UHMWPE-1, with and without the addition of 2 wt.% of DWCNTs, were compared. The results of this comparison are presented in Figure 17, which shows the tensile strength depending on the overall deformation degree.

Figure 17 illustrates that even at high overall deformation ratios, the discrepancy between the orientation strengthening of composites filled with NPs and that of non-filled UHMWPE is minimal. This result supports the conclusions drawn from the data presented in Figure 12b, Figure 13 and Figure 16. This allows for the production of highly electrically conductive, ultra-strong composite tapes that possess all the distinctive properties of UHMWPE. In particular, the composite tapes modified with 2 wt.% of DWCNTs and with an overall deformation ratio value of ~76 were found to have a tensile strength of ~3.3 GPa and a resistivity of ~500 Ohm*cm. It is anticipated that further optimization of the processing parameters will lead to even more enhanced electrophysical characteristics of the UHMWPE-based materials.

#### 3.3.3. SEM of the Highly Oriented Composite Tapes

**Figure 18:**
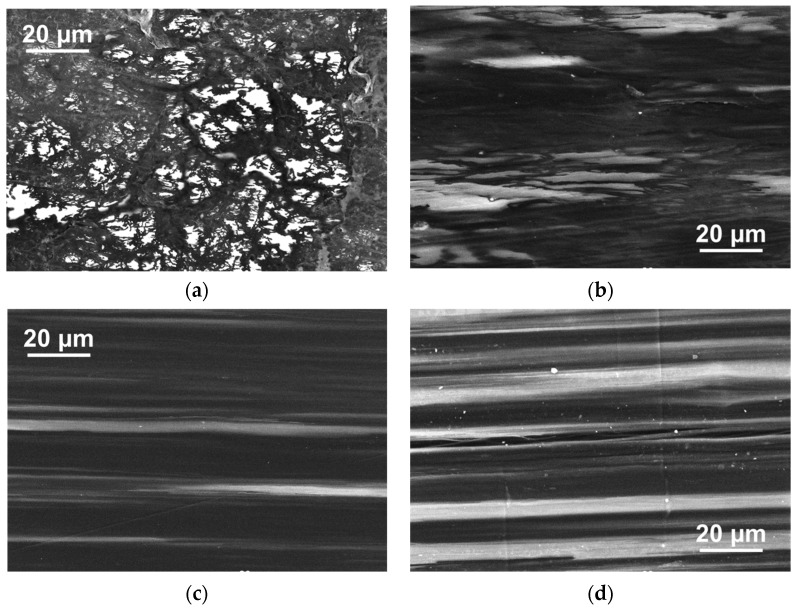
SEM images of the surfaces of composites based on UHMWPE-1 filled with 2 wt.% of DWCNTs, characterized by the combined (homogeneous shear deformation followed by uniaxial deformation) deformation ratio of (**a**) 1, (**b**) 5, (**c**) 38, or (**d**) 76.

To gain insight into the morphological changes occurring in UHMWPE-based composite materials filled with DWCNTs during orientation deformation, an attempt was made to visualize these changes up to high degrees of deformation. The low-temperature cleavage approach, which has previously been employed to visualize the filler distribution in composites deformed to a deformation ratio (Figure 11), was not feasible in this instance due to the high strength of the highly oriented state. Concurrently, the SEM of the tape surfaces of the non-filled UHMWPE exhibited uniform images that did not reflect the grain-like structure of the material. Fortunately, the distribution of highly electrically conductive NPs along the boundaries of UHMWPE grains enabled the tracking of morphological evolution during deformation. The contrasting results of the structural elements of UHMWPE using SEM for composites based on UHMWPE-1 filled with 2 wt.% DWCNTs and characterized by the different deformation ratio values are presented in Figure 18.

As illustrated in Figure 18, the UHMWPE grains, with a characteristic size of several microns in the non-deformed state (Figure 18a), demonstrated a notable degree of orientation as the deformation ratio increased. The aspect ratio of the UHMWPE grains appeared to correlate with the deformation ratio, particularly for low values of the ratio (Figure 18b). For the high values of the deformation ratio (Figure 18d), this may be less reliable due to the potential for material continuity to be broken in the direction transverse to the orientation. This may be attributed to the UHMWPE fibrillation process. The findings demonstrate that the distinctive nature of the highly segregated structure allows for the acquisition of data regarding the average size and aspect ratio of the UHMWPE macroscopic structure elements during the orientation deformation process by directly examining the surface of the tapes.

### 3.4. Temperature Dependences of the Composite Characteristics

A study was conducted to investigate the temperature dependencies of the electrical conductivity of compacted as well as deformed UHMWPE-based composite materials under homogeneous shear conditions. The findings of this study have enabled the formulation of conclusions regarding the mechanisms of interaction between the polymer component and the segregated electrically conductive network formed through coating d-UHMWPE RPs with NPs and subsequent solid-state processing of the mixture.

#### 3.4.1. Thermal Treatment of the Compacted Composites

**Figure 19:**
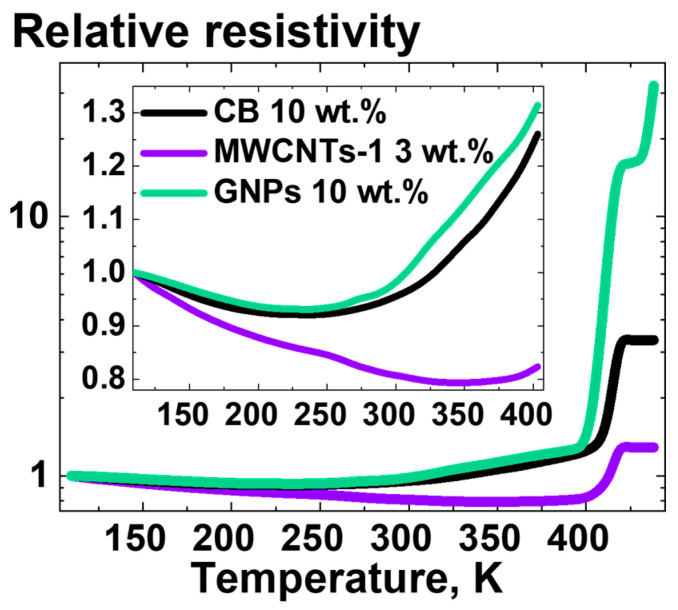
Relative resistivity of the composites based on UHMWPE-1 filled with 10 wt.% of CB, 3 wt.% of MWCNTs-1, or 10 wt.% of GNPs as a function of temperature. The inset depicts the same graph without the high-temperature (above 400 K) region. The relative resistivity was calculated by dividing the resistivity value for a specific temperature by the resistivity value corresponding to the lowest studied temperature (110 K).

Figure 19 presents the results of the measurements of the relative resistivity of the composites based on UHMWPE-1 filled with 10 wt.% of CB, 3 wt., 10 wt.% of GNPs, or of MWCNTs-1 as a function of temperature.

As shown in the inset of Figure 19, for all tested composite samples, the resistivity dependence on the temperature in the low-temperature region (below 400 K) was characterized by an initial decrease followed by a final increase. This was attributed to the two competing processes occurring in the composite material: the thermal expansion of the polymer matrix and the conductivity increase in the NP-enriched layers between the polymer grains due to the tunneling effect. It can be postulated that thermal expansion exerts an influence on the resistivity of the conducting network that is analogous to that of uniaxial strain (Figure 15).

At temperatures approaching the melting point of UHMWPE, a notable increase in resistivity was observed for all composite samples under study (Figure 19). This can be attributed to the sharp volume increase in the polymer phase during the melting process. Concurrently, the NPs are becoming capable of entering the volume of UHMWPE grains, thereby reducing the degree of segregation of the composite structure. This is evident from the loss of conductivity of the composite samples cooled after melting compared to the conductivity of the cold-pressed samples.

#### 3.4.2. Annealing of the Oriented Composites

**Figure 20:**
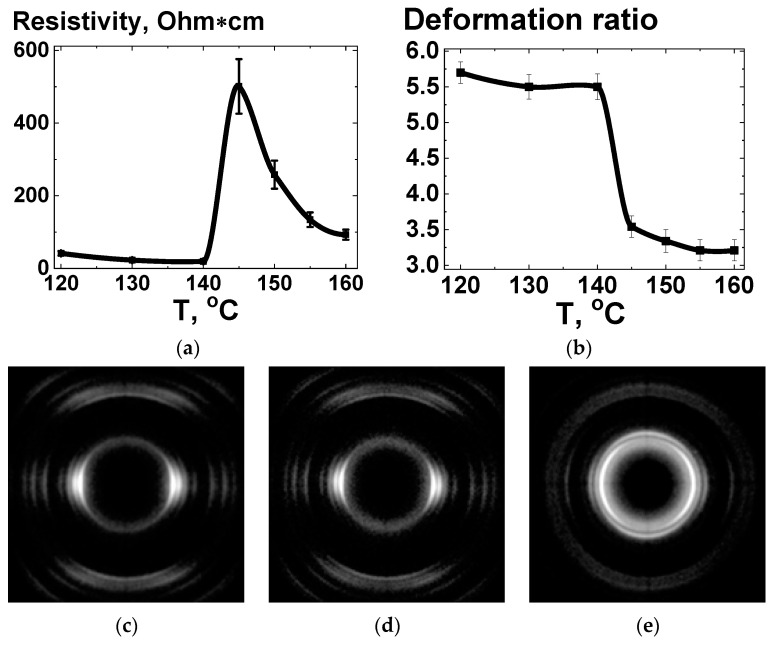
Dependencies of (**a**) specific resistivity and (**b**) deformation ratio on the annealing temperature for composite samples based on UHMWPE-1 filled with 10 wt.% of GNPs; (**c**,**d**) X-ray diffraction patterns of composite samples based on UHMWPE-1 filled with 10 wt.% of GNPs after subjecting them to the deformation under homogeneous shear conditions with a ratio of ~6. The diffraction patterns are obtained after annealing the composites at (**c**) 120, (**d**) 140, and (**e**) 145 ^°^C (see Section 2.3).

The effect of annealing on the conductivity and structure of UHMWPE-based composites subjected to homogeneous shear deformation was examined. Figure 20 shows dependencies of resistivity (Figure 20a) and deformation ratio (Figure 20b) on the annealing temperature of composites comprising UHMWPE-1 and GNPs, subjected to deformation with a ratio of ~6.

As shown in Figure 20, the annealing temperature had a negligible impact on the electrical conductivity (Figure 20a) and deformation ratio (Figure 20b) of UHMWPE at temperatures below its melting point (~141.5 °C). Upon reaching the melting temperature, a reduction in the dimensions of the oriented sample was observed (Figure 20b). However, the shrinkage ceased at a deformation ratio of ~3. Additionally, the XRD data (Figure 20e) indicated the presence of residual orientation. Upon reaching a deformation ratio of ~3 at temperatures above 145 °C, no further discernible changes were observed in the deformation ratio (Figure 20b) or in the XRD patterns. It is known that the melting of the solid-state processed materials, based on the d-UHMWPE RP, results in the entanglement of polymer chains, leading to the formation of a rubber-like material state [32]. This phenomenon may contribute to the retention of the initial orientation in the material.

Following the overcoming of the melting point, a notable increase in the resistivity of the material was observed (Figure 20a). It can be reasonably inferred that the cause of the resistivity increase is the same as that responsible for the conductivity loss in the compacted composite samples, as illustrated in Figure 19. The subsequent annealing process resulted in the restoration of the conductive network at higher temperatures. The degree of conductivity restoration was dependent on the annealing temperature, which influenced the mobility of the NPs. It can be observed that even at the highest applied temperatures the conductivity of the samples remained below the conductivity value prior to the melting. Given that higher conductivity levels are anticipated at lower deformation ratio values (Figure 12a), the decline in the conductivity can be attributed to the loss of the extreme character of the filler segregation in the composite volume.

### 3.5. Direct Extrusion of the Mixtures of d-UHMWPE RP and NPs

**Figure 21:**
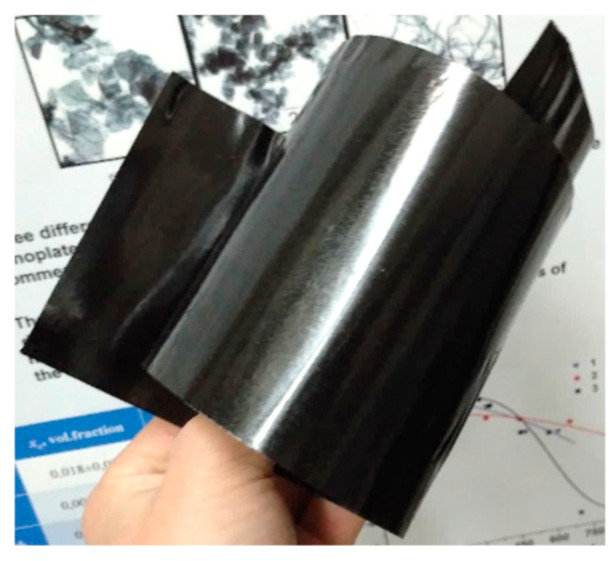
Photograph of the oriented tape obtained through the direct introduction of the mixture of UHMWPE-1 RP and 3 wt.% of MWCNTs-1 into the hot zone between the rotating rollers.

In order to ascertain whether the processing procedure could be simplified by combining two stages—compactization and homogeneous shear deformation—an attempt was made to process the d-UHMWPE RP and NPs mixtures by directly introducing them into the hot zone between the rotating rollers. Figure 21 shows the image of the resulting tape obtained from the mixture of UHMWPE-1 RP and 3 wt.% of MWCNTs-1.

By measuring the thickness of the resulting tapes and conducting an XRD analysis, it was determined that the resulting orientation degree for the composite material depends on the type and the content of the filler used. The composite tapes modified with GNPs exhibited the highest degree of deformation, while tapes modified with CB demonstrated a relatively low final orientation. In particular, the composite tapes filled with 10 wt.% of GNPs exhibited a deformation ratio of ~4, while the deformation ratio for tapes filled with 10 wt.% of CB was ~2.5.

A significant limitation of this methodology is the considerable defectiveness of the resulting tapes along their edges, which results in a substantial loss of material when the edges are cut. Additionally, the inconsistency in the deformation ratio for composites filled with NPs of varying types and content values presents a challenge in standardizing the processing procedure for accurate comparison of the structure and properties of the composites.

The one-stage approach, however, enables the effective scaling of the production of highly oriented composite tapes. By examining the deformation behavior and conductivity of the highly segregated electrically conductive networks processed in a two-stage manner, it is possible to anticipate the characteristics of the materials obtained from the direct processing of powder mixtures. This approach is particularly relevant given that the ultimate characteristics of highly oriented solid-state processed UHMWPE-based materials are not dependent on the ratio of deformation under homogeneous shear conditions, but rather on the overall deformation ratio. Consequently, the one-stage manufacturing approach can be considered the most feasible for the industrial-level production of such materials.

## 4. Conclusions

This work demonstrates the feasibility of producing composite materials with an extreme segregation of the filler within the polymer matrix. This was achieved through the solid-state processing of mixtures comprising nascent d-UHMWPE and NPs of varying types. The necessity for a meticulous consideration of component selection and processing parameters to obtain highly conductive composites based on solid-state processed d-UHMWPE RP was demonstrated. It was shown that, despite the potential for obtaining highly oriented and strong tapes from d-UHMWPE RPs of varying syntheses due to the distinctive structural characteristics of the RP, the structural parameters enabling the successful coating of an RP particle with functional NPs can be entirely different. Additionally, it was established that a tailored approach is essential for optimizing the distribution of particles on the surface of d-UHMWPE RP particles, with each filler type ideally requiring a unique strategy.

It was shown that the deformation under homogeneous shear conditions represents an effective method for enhancing the mechanical properties of electrically conductive composites based on UHMWPE while simultaneously overcoming the brittle fracture barrier inherent to the cold-compacted materials. The orientation strengthening efficiency for such composites, which are distinguished by an extremely segregated structure, was proven to be independent of the type and the content of the filler, even at high filler content values. Concurrently, the orientation strengthening is invariably accompanied by a consistent decline in the electrical conductivity of the composites. This phenomenon can be partially offset by the use of NPs of an exceptionally long length, such as CNTs. In particular, the effectiveness of utilizing a particular type of DWCNTs as a filler was demonstrated.

The uniaxial orientation of solid-state processed composites based on UHMWPE filled with DWCNTs, conducted at temperatures proximate to the melting point of UHMWPE, demonstrated equivalence to homogeneous shear deformation with regard to impact on electrical conductivity and orientation strengthening. Therefore, the initial stage of homogeneous shear deformation can be employed to achieve any desired deformation ratio, thereby overcoming the brittle nature of the material. Subsequently, uniaxial deformation can be utilized to obtain highly oriented, ultra-strong UHMWPE composite tapes. The resulting materials have the potential to be utilized in a multitude of practical applications across a wide range of industrial sectors.

The investigation into the impact of temperature and annealing on the characteristics of solid-state compacted and deformed UHMWPE-based composites has revealed the duality inherent in the highly segregated structure of these composites. In particular, it can be observed that the conductive network comprises two distinct structural levels: a highly dense packing of NPs at the surface of the UHMWPE grains and a honeycomb-like structure of polymer grains separated by thin NP layers. As a result, temperature affects the conductive structure through a variation in the temperature dependencies of the NP contact resistances and a change in the geometry of the filler distribution due to the thermal expansion of the polymer grains. Furthermore, the compacted and preliminary deformed composite samples exhibited a reduction in conductivity due to the loss of the extreme segregation of the filler following the transition to the melted state of the material. It was demonstrated that the shrinkage observed in this case does not result in a reduction of the resistivity, contrary to expectations. This suggests the existence of structural and deformational differences in the conductive network of UHMWPE-based composites processed in the solid and melted states.

The capacity of the d-UHMWPE RP and NPs mixtures to be processed directly into oriented tapes, which can subsequently be oriented further to achieve a high-strength state, allows for the production of materials at a scale suitable for a multitude of industrial-level applications.

In conclusion, the results of the comprehensive study of the solid-state processing parameters for obtaining composites based on nascent d-UHMWPE RP, as well as their structure and properties, demonstrate a distinctive character of the extremely segregated structure of the composites. It is possible to alter the distribution of fillers for the studied method of processing and matrix polymer by modifying the processing parameters and the type and quantity of the filler. Concurrently, the deformational behavior of the polymer matrix is independent of the presence of the filler, allowing for the study of the effect of orientation on the segregated filler network under identical conditions. This makes the composites exhibiting this structural configuration a viable model system for analytical and numerical investigations of the relationships between the filler type/structure, composite structure, and composite properties.

## Data Availability

The raw data supporting the conclusions of this article will be made available by the authors on request.
